# *MACC1* expression levels as a novel prognostic marker for colorectal cancer

**DOI:** 10.3892/ol.2014.2460

**Published:** 2014-08-19

**Authors:** HIROFUMI YAMAMOTO, NORIKATSU MIYOSHI, KOSHI MIMORI, TOSHIKI HITORA, MASAYOSHI TOKUOKA, SHIKI FUJINO, HALEY L. ELLIS, HIDESHI ISHII, SHINGO NOURA, MASAYUKI OHUE, MASAHIKO YANO, YUICHIRO DOKI, MASAKI MORI

**Affiliations:** 1 Department of Gastroenterological Surgery, Osaka University Graduate School of Medicine, Suita, Osaka 565-0871, Japan; 2 Department of Surgery, Osaka Medical Center for Cancer and Cardiovascular Diseases, Nakamichi, Osaka 537-8511, Japan; 3 Department of Molecular and Cellular Biology, Division of Molecular and Surgical Oncology, Kyushu University, Medical Institute of Bioregulation, Beppu, Osaka 874-0838, Japan; 4 Department of Surgery, Osaka Rosai Hospital, Sakai, Osaka 591-8025, Japan; 5 Department of Surgery, Yao Municipal Hospital, Yao, Osaka 581-0069, Japan; 6 Human Oncology and Pathogenesis Program, Memorial Sloan-Kettering Cancer Center, New York, NY 10065, USA; 7 Department of Frontier Science for Cancer and Chemotherapy, Osaka University, Yamadaoka, Suita, Osaka 565-0871, Japan

**Keywords:** metastasis-associated in colon cancer-1, prognosis, colorectal cancer

## Abstract

Metastasis-associated in colon cancer-1 (*MACC1*) is key in promoting tumor proliferation and invasion, and is mediated by the hepatocyte growth factor (*HGF*) and mesenchymal-epithelial transition factor. Previous reports have revealed that *MACC1* is a novel oncogene that is expressed in various types of gastrointestinal cancer. The present study comprised of 174 patients who underwent curative surgery for colorectal cancer (CRC). The correlation between gene expression and clinical parameters of the patients was assessed. It was identified that patients exhibiting high *MACC1* expression levels were statistically more susceptible to distant metastases and a poor prognosis, and those exhibiting low *MACC1* expression showed improved disease-free and overall survival than those with high expression. Therefore, the present data indicates that *MACC1* expression levels may present as a prognostic factor in CRC patients.

## Introduction

In developed countries where the aging population is increasing, cancer is one of the most prominent diseases with regard to public welfare and health measures. One in four mortalities in the USA, for example, is due to cancer (1). In the USA, the incidence of colorectal cancer (CRC) has increased significantly in recent years due to the changing lifestyle of the population and is currently one of the most frequently exhibited malignancies and leading causes of cancer-related mortality. The metastatic dissemination of primary tumors is directly associated with patient survival, and distant metastases, such as of the liver or lungs, are the major cause of mortality in CRC patients. Furthermore, metastatic disease is the most frequent reason for treatment failure (2). Therefore, the identification of genes that are responsible for the development and progression of CRC, and comprehension of the clinical significance of these genes is critical for the diagnosis and adequate treatment of CRC. The characterization of these key molecules is promising for the development of novel treatment strategies for CRC.

The hepatocyte growth factor (*HGF*)/mesenchymal-epithelial transition factor (*MET*) signaling pathway was reported to have a key role in cellular growth, invasiveness and metastasis (3–5). The metastasis-associated in colon cancer-1 (*MACC1*) induces *MET* expression and promotes *HGF*-induced scattering, which the mitogen-activated protein kinase (*MAPK*) signaling pathway prevents (6). This indicates that *MACC1* expression in primary tumors is associated with metastasis and results in a poor prognosis.

The aim of the present study was to analyze the correlation between *MACC1* expression levels, in tissue obtained from CRC patients, with their clinicopathological factors, and to investigate the possible functions of *MACC1* in the metastasis of CRC.

## Materials and methods

### Clinical tissue samples

One hundred and seventy-four patients (99 males and 75 females) with CRC underwent curative surgery of CRC and distant metastases (if present) at the Department of Gastroenterological Surgery, Osaka University Graduate School of Medicine and Medical Institute of Bioregulation at Kyusyu University (Osaka, Japan) between 1994 and 2003. No patients had received chemotherapy or radiotherapy prior to surgery. Primary CRC specimens and adjacent normal colorectal mucosa were obtained from patients following receipt of written informed consent, which was in accordance with the institutional ethical guidelines. The surgical specimens were fixed in formalin, processed through graded ethanol, and embedded in paraffin. The sections were stained with hematoxylin and eosin and Elastica van Gieson stain (Merck Millipore, Billerica, MA, USA), and the degree of histological differentiation, lymphatic invasion, and venous invasion was examined. Additionally, samples of each specimen were frozen in liquid nitrogen immediately after resection and stored at −80°C until RNA extraction. Following surgery, the patients underwent follow-up blood examinations to assess the tumor markers, serum carcinoembryonic antigen and cancer antigen 19–9 and imaging, such as abdominal ultrasonography, computed tomography and chest X-rays were conducted every 3–6 months. Postoperatively, stage III and IV patients received 5-fluorouracil-based chemotherapy [mFOLFOX6; 85 mg/m^2^ oxaliplatin and 2800 mg/m^2^ 5-fluorouracil, for two weeks for a total of 12 courses of treatment; 300 mg/m^2^/day UFT, for 28 days for five weeks for five courses of treatment; 2500 mg/m^2^/day capecitabine for 14 days for three weeks for 8 courses of treatment, and/or 80 mg/m^2^/day TS-1 (tegafur, gimestat and otastat potassium) for 28 days for six weeks and four courses of treatment]. Adjuvant therapeutic strategies were performed, except for stage I and II patients who received no chemotherapy, according to the guidelines laid out by the Japanese Society for Cancer of the Colon and Rectum (7). Clinicopathological factors were assessed according to the tumor node metastasis (TNM) classification system of the International Union Against Cancer (8). This study was approved by the ethics committee of Osaka University Graduate School of Medicine (Osaka, Japan).

### RNA preparation and expression analysis

Total RNA was prepared using TRIzol reagent (Invitrogen Life Technologies, Carlsbad, CA, USA) or using DNase and a modified acid guanidinium-phenol-chloroform procedure (9). Reverse transcription (RT) was performed with SuperScript™ II (Invitrogen Life Technologies) or by the methods reported previously (10) and an *MACC1* fragment was amplified by polymerase chain reaction (PCR). Two human *MACC1* oligonucleotide primers were designed as follows: Forward, 5′-TTCTTTTGATTCCTCCGGTGA-3′ and reverse, 5′-ACTCTGATGGGCATGTGCTG-3′. A PCR kit (Takara Ex Taq; Takara Bio Inc., Shiga, Japan) on a GeneAMP^®^ PCR System 9600 (PE Applied Biosystems, Foster City, CA, USA) was used to perform 35 cycles of PCR with the following parameters: 95°C for 40 sec, 45°C for 40 sec and 72°C for 60 sec. An 8-μl aliquot of each reaction mixture was size-fractionated in a 1.5% agarose gel and visualized with ethidium bromide staining. To ensure the RNA was not degraded, a PCR assay with primers specific for the glyceraldehydes-3-phosphate dehydrogenase (*GAPDH*) gene was performed for 1 min at 95°C, 1 min at 56°C, and 1 min at 72°C for 30 cycles. The *GAPDH* primers were as follows: Forward, 5′-TTGGTATCGTGGAAGGACTCA-3′ and reverse, 5′-TGTCATCATATTGGCAGGTT-3′ and produced a 270-bp amplicon. Complementary DNA (cDNA) from Human Reference Total RNA (Clontech Laboratories, Mountain View, CA, USA) was analyzed concurrently and served as a positive control. For quantitative assessment, RT-qPCR was performed using a LightCycler^®^ FastStart DNA Master SYBR Green I kit (Roche Diagnostics, Tokyo, Japan) for cDNA amplification of *MACC1* and *GAPDH*. The amplification protocol consisted of 35 cycles of denaturation at 95°C for 10 sec, annealing at 60°C for 10 sec, and elongation at 72°C for 10 sec. The products were then subjected to a temperature gradient from 55°C to 95°C with continuous fluorescence monitoring to produce a melting curve of the products. The expression ratios of the *MACC1* mRNA copies in the tumor and normal tissues were calculated following normalization against the *GAPDH* mRNA expression.

### Statistical analysis

The association between *MACC1* expression and patient clinicopathological factors was analyzed using the χ^2^ test. Kaplan-Meier survival curves were plotted and compared with the generalized log-rank test. Univariate and multivariate analyses were performed to identify prognostic factors using a Cox proportional hazards regression model. The *in vitro* assay values were analyzed using the Wilcoxon signed-rank test. All tests were analyzed with JMP software (SAS Institute Inc., Cary, NC, USA) and P<0.05 was considered to indicate a statistically significant difference.

## Results

### Expression levels of MACC1 in clinical tissue specimens

**Figure 1:**
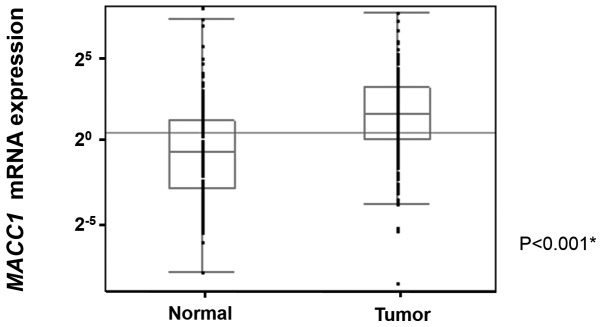
*MACC1* mRNA expression levels in clinical tissue specimens. Quantitative reverse transcription-polymerase chain reaction on 174 paired clinical samples showed that 143 cases (82.2%) exhibited higher levels of *MACC1* mRNA in tumors than in the paired normal tissues. The mean *MACC1* mRNA expression level in the tumor tissues (normalized to glyceraldehydes-3-phosphate dehydrogenase gene expression) was significantly different when compared with the corresponding normal tissues (P<0.001, Wilcoxon-signed rank test).

RT-qPCR analysis was performed on tissues from primary CRC and adjacent normal colorectal regions. *MACC1* expression was calculated by normalising it to *GAPDH* expression for each tumor or normal tissue sample (Fig. 1). A significant difference was identified between the tissue types, with the average expression in tumor tissues larger than that of the corresponding normal tissues. In the following analyses, *MACC1* expression, normalized to *GAPDH* expression, in tumor tissue was calculated following division by *MACC1*/*GAPDH* expression in the normal tissue.

### Expression levels of MACC1 and patient clinicopathological characteristics

For the clinicopathological evaluation, experimental samples were divided into two groups according to the expression status. Patients with values >1 (*MACC1* expression level of tumor tissue greater than that of the corresponding normal tissue) were assigned to the high-expression group and patients with values <1 were assigned to the low-expression group. The clinicopathological factors that were associated with the *MACC1* expression status of the 174 patients are summarized in Table I. The data indicates that the level of *MACC1* expression was not significantly correlated with the clinicopathological factors.

### Association between MACC1 expression and prognosis

**Figure 2:**
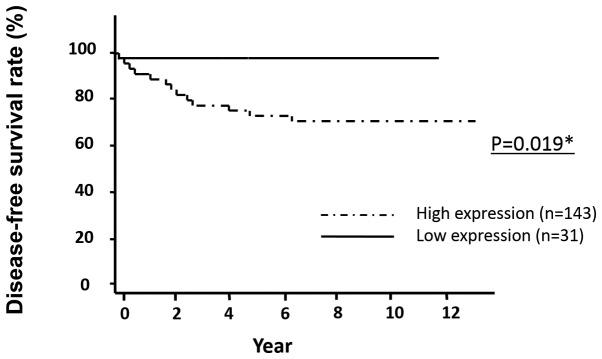
Disease-free survival curves based on the *MACC1* mRNA expression status of colorectal cancer patients following curative surgery. The postoperative disease-free survival rate was significantly lower in patients in the high-*MACC1* expression group compared with the low-expression group (P=0.019, log-rank test).

**Figure 3:**
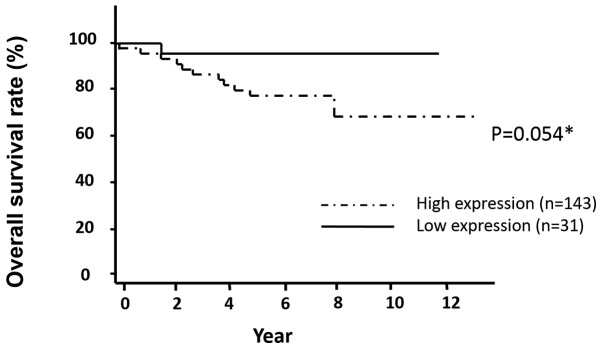
Overall survival curves based on the *MACC1* mRNA expression status of colorectal cancer patients. The postoperative overall survival rate was significantly lower in patients in the high-*MACC1* expression group compared with the low-expression group (P=0.054, log-rank test).

The data showed that the postoperative disease-free survival rate was significantly lower in patients in the high-expression group than that of the low-expression group (P=0.019; Fig. 2). The postoperative overall survival rate was lower in patients in the high-expression group than in the patients in the low-expression group (P=0.054; Fig. 3). The median follow-up was 4.1 years. Table II shows the results of the univariate and multivariate analyses for factors associated with disease-free survival. The univariate analysis showed that age (P=0.035), tumor invasion (P<0.001), lymph node metastasis (P<0.001), lymphatic invasion (P<0.001), venous invasion (P=0.011), distant metastasis (P<0.001) and *MACC1* expression (P=0.005) were significantly correlated with disease-free survival. The multivariate regression analysis indicated in the *MACC1* high-expression group (hazard ratio [HR], 2.27; 95% confidence interval [CI], 1.01–9.71; P=0.044), lymph node metastasis (HR, 3.15; 95% CI, 1.44–7.48; P=0.003), lymphatic invasion (HR, 2.87; 95% CI, 1.28–6.86; P=0.010) and distant metastasis (HR, 12.83; 95%; CI, 4.62–34.57; P<0.001) were independent predictors of disease-free survival.

## Discussion

*HGF* activates the *HGF*/*MET* signaling pathway, which is involved in metastasis of CRC. The *HGF* receptor, the gene for the receptor tyrosine kinase, *MET* was identified as the first transcriptional target of *MACC1* (11). *MACC1* is located on chromosome 7 and was identified through genome-wide expression analyses conducted on primary and metastatic colon cancer (12). *MACC1* binds to, and promotes, *MET* gene expression by translocating from the cytoplasm to the nucleus, leading to migration, invasion and metastasis.

The present study demonstrated that *MACC1* expression levels are an independent factor of disease-free survival for CRC. Tumor malignancy was identified to correlate with *MACC1* expression levels and *MACC1* expression may affect the values of other prognostic factors in multivariate analysis, such as distant metastasis, which was found to be significant in univariate analysis. *MACC1* expression levels were a significant prognostic factor, reflecting disease-free survival as well as the occurrence of distant metastasis. The present study is considered to be important as it has provided analyses of a large number of samples, which demonstrate that *MACC1* expression levels may be used as a statistically significant marker for CRC metastasis following curative resection, along with other reported predictors (13). The present results support recent reports that a *MACC1*-dependent signaling pathway is involved in the progression of CRC (12,14,15).

It is useful to determine the necessity for intensive follow-up and adjuvant CRC therapy by predicting recurrence and metastases following curative surgical resection (16–18). Certain patients respond well to the treatment of CRC, however others do not. Thus, more precise and personalized predictions and strategies for treating metastases are required (19). In the present study, the clinicopathological analysis of a large number of patients revealed that CRC samples exhibiting a low expression of *MACC1* were an improved predictor for disease-free and overall survival when compared with the high-expression group. The data indicates that *MACC1* expression levels are an effective predictor of CRC prognosis.

In CRC, various adjuvant chemotherapeutic strategies are facilitative during certain disease stages and indicate the usefulness of less invasive surgery for CRC (13,16–18,20–26). For these cases, an informative prognostic marker, which is independent from the traditional TNM classification and contributes to diagnosis and treatment, is important. In conclusion, the present data indicates *MACC1* expression levels as a potential prognostic marker for CRC. Whilst improved preoperative and postoperative treatment strategies for CRC, such as chemo- and radiotherapy combined with surgery, have contributed to the reduction of recurrences, eventually half of the cases metastasize despite the systemic chemotherapy and surgery (27). Adjuvant chemotherapy for CRC is advantageous in cases where recurrence is considered to be likely. In these cases, *MACC1* expression level analysis may present as a tool to predict poor prognosis and provide adequate treatment for patients.

## Figures and Tables

**Table I:** Clinicopathological factors and *MACC1* mRNA expression in 174 colorectal cancer tissue samples.

Factor	Low expression n=31 (%)	High expression n=143 (%)	P-value
Age (years)			
<68	19 (61.3)	70 (48.9)	0.212
≥68	12 (38.7)	73 (51.1)	
Gender			
Male	13 (41.9)	86 (60.1)	0.063
Female	18 (58.1)	57 (39.9)	
Histological grade			
Wel-Mod	31 (100)	134 (93.7)	0.151
Por-Muc	0 (0)	9 (6.3)	
Tumor size			
<30 mm	11 (35.5)	41 (28.7)	0.452
≥30 mm	20 (64.5)	102 (71.3)	
Tumor invasion			
Tis	7 (22.6)	8 (5.6)	0.006^a^
T1	4 (12.9)	12 (8.4)	
T2	8 (25.8)	23 (16.1)	
T3	9 (29.0)	74 (51.7)	
T4	3 (9.7)	26 (18.2)	
Lymph node metastasis			
N0	23 (74.2)	83 (58.0)	0.094
N1-2	8 (25.8)	60 (42.0)	
Lymphatic invasion			
Absent	17 (54.8)	83 (58.0)	0.743
Present	14 (45.2)	60 (42.0)	
Venous invasion			
Absent	27 (87.1)	111 (77.6)	0.237
Present	4 (12.9)	32 (22.4)	
Distant metastasis			
M0	31 (100)	134 (93.7)	0.151
M1	0 (0)	9 (6.3)	

The tumor node metastasis classification of the International Union Against Cancer (8) was used to determine the extent of tumor invasion, lymph node metastasis and distant metastasis. Wel, well-differentiated adenocarcinoma; Mod, moderately differentiated adenocarcinoma; Por, poorly differentiated adenocarcinoma; Muc, mucinous adenocarcinoma; Tis, carcinoma *in situ*.

a P<0.05 indicates a statistically significant value.

**Table II:** Univariate and multivariate analyses for disease-free survival (Cox proportional hazards regression model).

	Univariate analysis			Multivariate analysis		
		
Factor	HR	95% CI	P-value	HR	95% CI	P-value
Age (years)						
(<68/≥68)	2.10	1.05–4.49	0.035	1.99	0.98–4.28	0.056
Gender						
(Male/Female)	1.46	0.79–3.12	0.289			
Histological grade						
(Por-Muc/Wel-Mod)	1.52	0.61–2.79	0.304			
Tumor size (mm)						
(≥30/<30)	1.38	0.93–2.18	0.106			
Tumor invasion						
(T3-4/Tis-2)	7.11	2.53–29.64	<0.001^a^	1.69	0.52–7.62	0.403^a^
Lymph node metastasis						
(N1-2/N0)	5.87	2.83–13.35	<0.001^a^	3.15	1.44–7.48	0.003^a^
Lymphatic invasion						
(Present/Absent)	3.71	1.85–7.94	<0.001^a^	2.87	1.28–6.86	0.010^a^
Venous invasion						
(Present/Absent)	2.62	1.25–5.21	0.011^a^	2.24	0.98–5.06	0.054
Metastasis						
(M1/M0)	10.18	4.25–21.95	<0.001^a^	12.83	4.62–34.57	<0.001^a^
*MACC1* expression						
(High/Low)	2.736	1.26–11.53	0.005^a^	2.27	1.01–9.71	0.044^a^

Clinicopathological factors, including tumor invasion, lymph node metastasis and distant metastasis were assessed according to the tumor node metastasis classification of the International Union Against Cancer (8). HR, hazard ratio; CI, confidence interval; Por, poorly differentiated adenocarcinoma; Muc, mucinous adenocarcinoma; Wel, well differentiated adenocarcinoma; Mod, moderately differentiated adenocarcinoma; Tis, carcinoma *in situ*; *MACC1*, metastasis-associated in colon cancer-1.

a P<0.05 indicates a statistically significant value.
